# Sclerochoroidal calcification associated with chondrocalcinosis: A clinical case

**DOI:** 10.1016/j.amsu.2022.103275

**Published:** 2022-01-29

**Authors:** O. Nabih, H. Hamdani, L. EL Maaloum, B. Allali, A. EL kettani

**Affiliations:** Medical Resident at Pediatric Ophthalmology Department, Ho^pital 20, Aou^t, 1953, Casablanca, Morocco

**Keywords:** Sclerochoroidal calcification, Bilateral calcifications, Chondrocalcinosis, Idiopathic calcifications, Indocyanine green angiography

## Abstract

**Introduction:**

Sclerochoroidal calcifications is a rare condition corresponding to senile plaques due to the deposition of calcium and phosphate in the sclera and choroid. It is an elderly patient's pathology. In most cases, it is idiopathic. However, some patients have systemic disorders leading to disturbances in phosphocalcic metabolism, which are at the origin of this disorder. A check-up is therefore necessary to detect them.

**Observation:**

We report the case of a 65 year old patient admitted to the ophthalmology department for a renewal of optical correction. The patient is known to have articular chondrocalcinosis on anti-inflammatory medication. Fundus examination revealed multiple white supra macular choroidal lesions with tumour-like appearance in both eyes. The ultrasound perfomed showed the calcic nature of the lesions. Fluorescein and indocyanine green angiograms showed no sign of activity or presence of neovessels.

A complete metabolic work-up, mainly phosphocalcic, was ordered again and the systemic diagnosis of articular chondrocalcinosis was retained.

**Discussion:**

Shields et al. first described, in 1997, a case of sclerochoroidal calcification in a patient with chondrocalcinosis and a normal metabolic profile. Few more have been added to the literature. It is generally considered to be predominantly bilateral and is most often seen as yellowish lesions. Two types of calcifications have been described to date, the plaque type and the pseudotumor type. Given the asymptomatic presentation, a fundus examination should be performed in patients with chondrocalcinosis. Generally, no treatment is necessary.

**Conclusion:**

Sclerochoroidal calcifications usually manifests as multiple discrete yellow placoid lesions in elderly asymptomatic patients. Visual prognosis for sclerochoroidal classification is good since the lesions tend to be away from the macula. They should not be confused with choroidal metastasis or achromic melanoma which require more extensive treatment.

## 1 Introduction

Sclerochoroidal calcifications is a rare condition corresponding to senile plaques due to the deposition of calcium and phosphate in the sclera and choroid [1].

They occur in elderly people with no predominance of either women or men.

They are characterised by buff-white sub-retinal lesions located preferentially in the supra-temporal region between the vascular arches and the equator.

These sclerochoroidal calcifications may be secondary to metabolic disturbances [2] or idiopathic [3].

Despite the well-known features, this condition is often misdiagnosed as choroidal metastasis, nevus, melanoma, or osteoma, hence the need for knowledge of this pathology.

We describe a rare case of sclerochoroidal calcifications associated with a known articular chondrocalcinosis.

This study has been reported in accordance with the SCARE criteria [4].

## 2 Clinical case

We report the case of a 65 years old patient admitted to the ophthalmology department for a renewal of optical correction.

The patient had been treated in the rheumatology department for articular chondrocalcinosis under anti-inflammatory and analgesic medication without any particular therapeutic project.

**Fig. 1:**
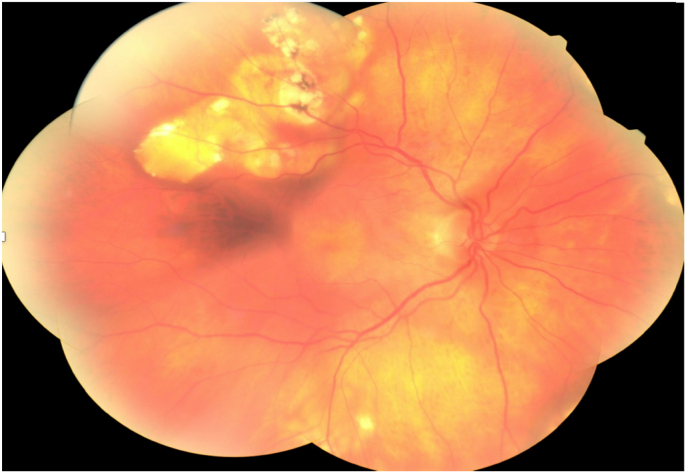
Mosaic retinography of the right eye showing multiple white supra-macular choroidal lesions with a pseudo tumoral appearance along the superior temporal arch.

**Fig. 2:**
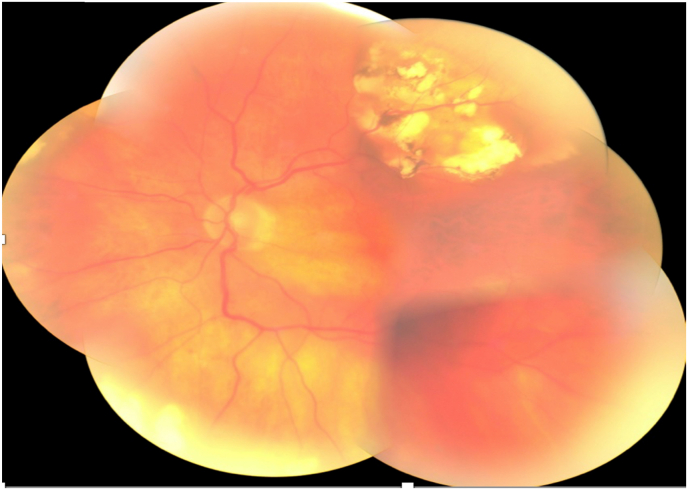
Mosaic retinography of the left eye showing a large white sessile supra macular choroidal lesion with a pseudo tumorous aspect.

The ophthalmological examination showed a corrected visual acuity of 8/10 in the right eye and 9/10 in the left eye. The slit lamp examination showed a non-disturbing bilateral nascent cortical cataract and normal eye tone in both eyes. Fundus examination revealed multiple white supra macular choroidal lesions with tumour-like appearance in both eyes [Fig. 1] [Fig. 2].

**Fig. 3:**
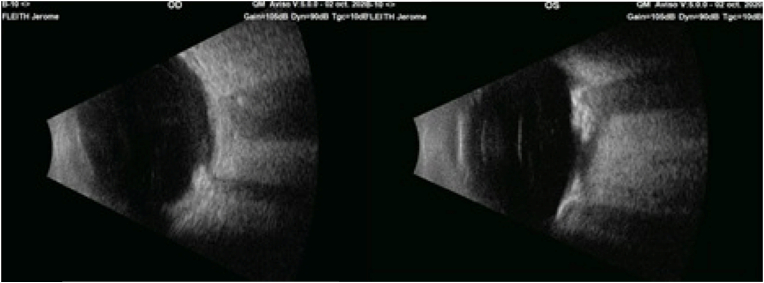
Ultrasound image showing calcified masses with posterior shadow cone.

An ultrasound scan was performed confirming the calcified nature of the lesions with a posterior shadow cone appearance [Fig. 3].

On fluorescein angiography, the lesions spared the macular area in both eyes.

**Fig. 4:**
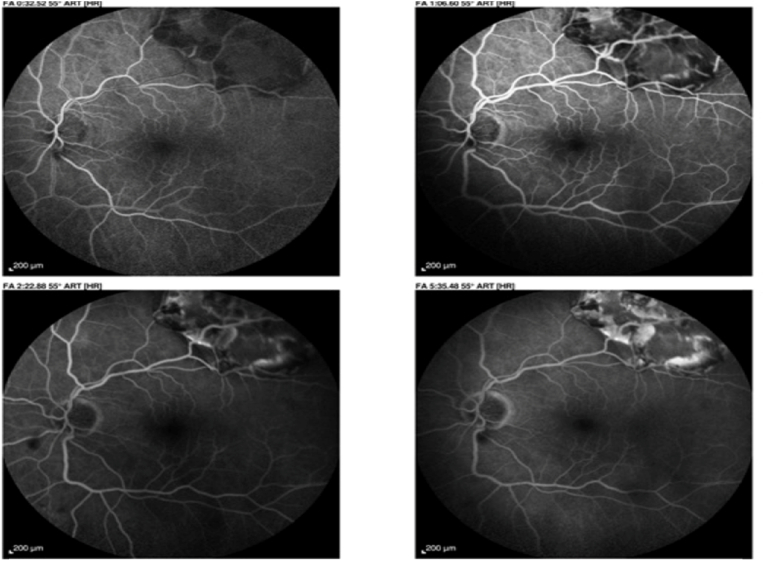
Fluorescein angiograms of the left eye at different times showing choriocapillaris permeation with mild hyperfluorescence at the late phase.

**Fig. 5:**
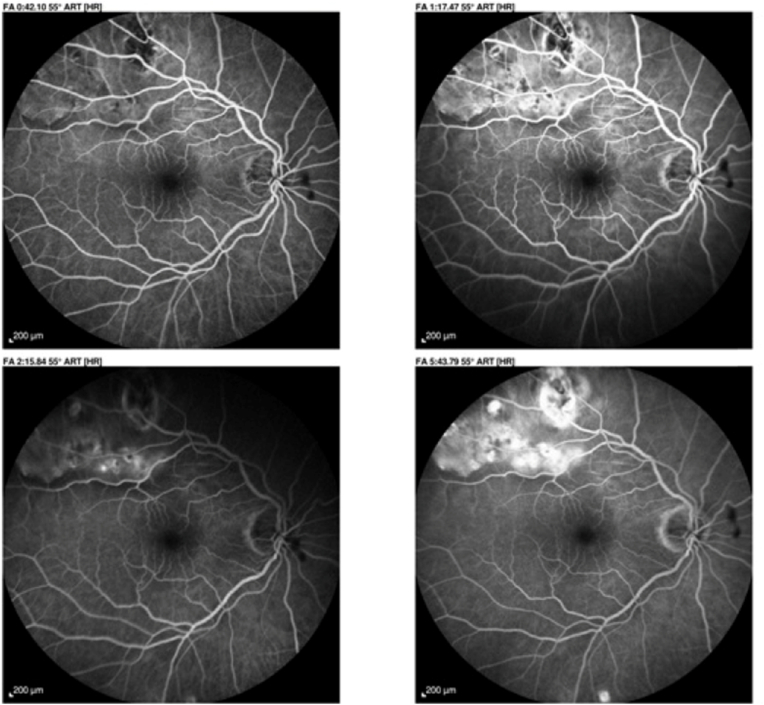
Fluorescein angiography of the left eye at different times showing anarchic choriocapillaris impregnation with hyperfluorescence in late phase.

They showed a hypofluorescent edge in the early sequence, but the choriocapillaris soaked normally and took on a normal dappled appearance, even slightly hyperfluorescent at the end of the sequence. No diffusion or pinpoint phenomena are visible at later sequences [Fig. 4] [Fig. 5].

**Fig. 6:**
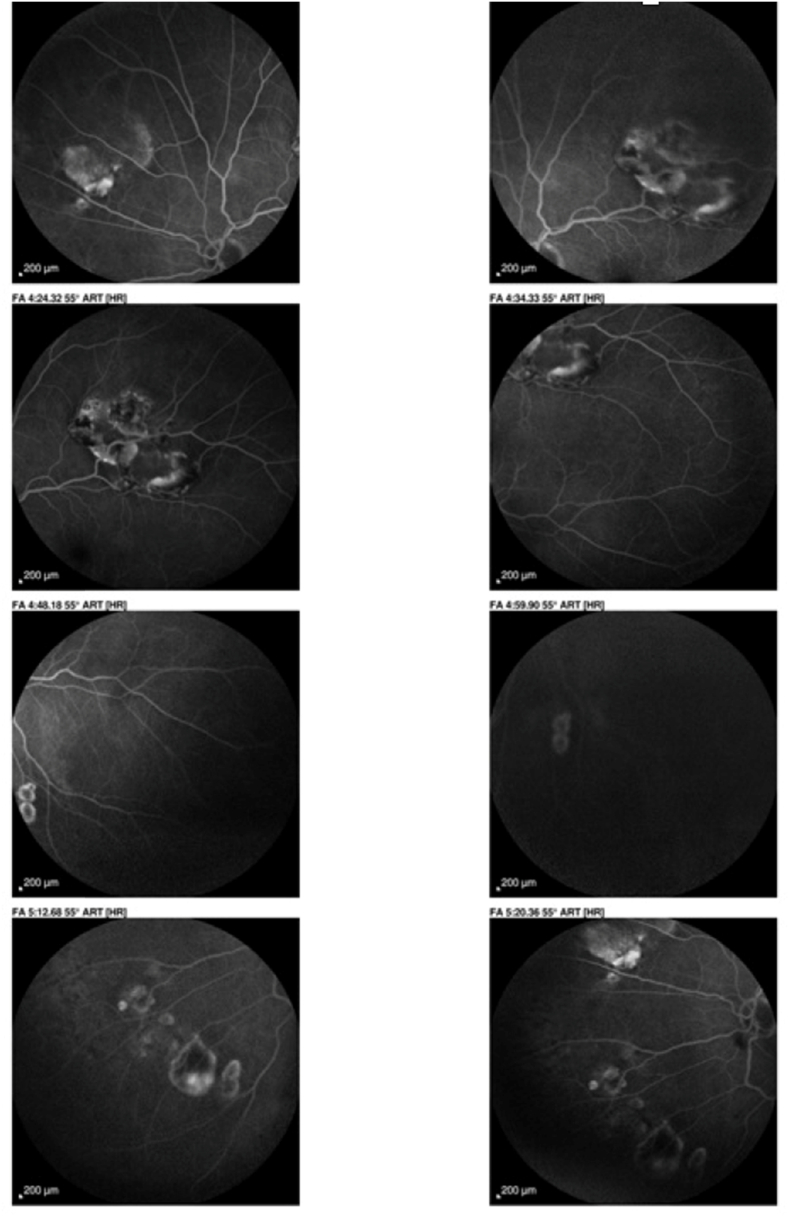
Indocyanine green angiography of the right eye showing hypocyanescent vessels in front of the choroidal mass with a slight impregnation at the late sequence.

**Fig. 7:**
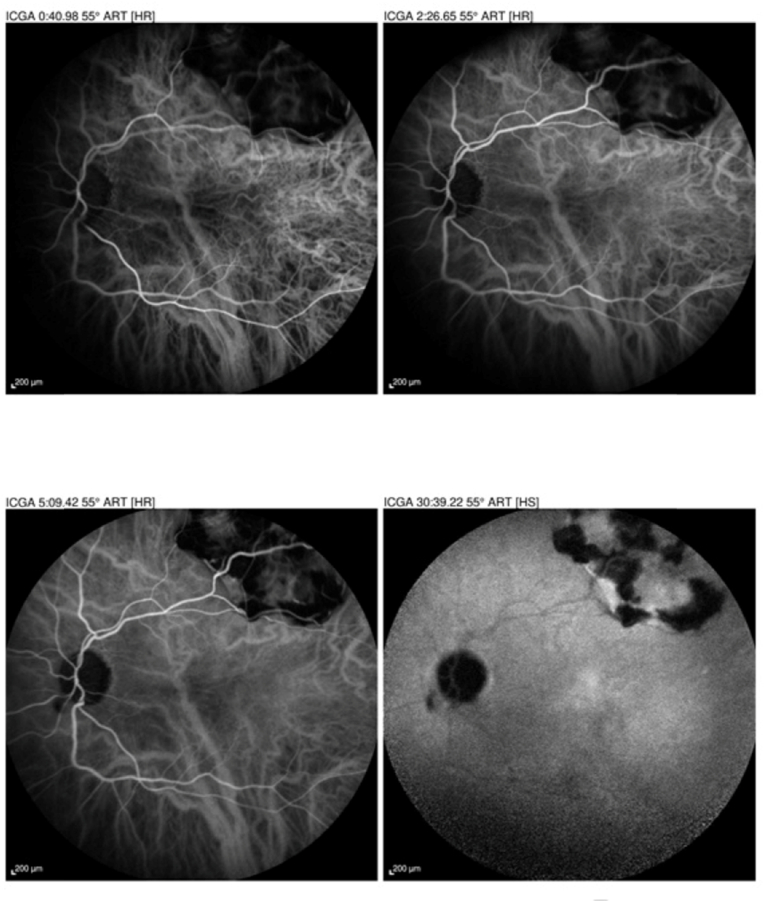
Indocyanine green angiography showing hypercyanotic vessels in front of the choroidal mass.

The lesion is slightly hypofluorescent on indocyanine green angiography, but the choroidal vascularity is normal at early, middle and late stages [Fig. 6] [Fig. 7].

**Fig. 8:**
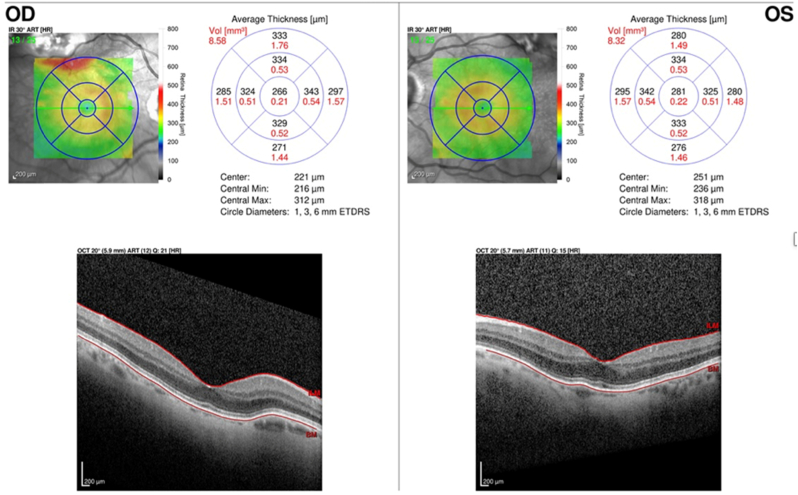
Optical coherence tomography image showing an elevation of the pigmentary epithelium more visible on the right with a thickening of the choriocapillaris.

Optical coherence tomography showed a normal retina and pigment epithelium but elevated especially on the right [Fig. 8].

A complete metabolic work-up, mainly phosphocalcic, was ordered again and the systemic diagnosis of articular chondrocalcinosis was retained.

A family screening of the children and siblings was carried out but no ocular involvement was found.

## 3 Comments

In 1997, Shields et al. [5] described a case of sclerochoroidal calcification in a patient with chondrocalcinosis and a normal metabolic profile.

In this report we describe an isolated case of sclerochoroidal calcifications associated with articular chondrocalcinosis. The inheritance of sclerochoroidal calcifications has never been described; however, hereditary forms of chondrocalcinosis have already been reported.

In our case, the patient had a 12-month follow-up showing no growth of calcifications and no progression to the macular area.

Two types of calcifications have been described to date, the plaque type and the pseudotumor type [6].

No study has shown the progression from plaque lesions to tumour-like lesions.

In 1992, Schachat et al. [7] reported 10 cases with a follow-up ranging from 7 months to 10 years, in which no evolution of the lesion was observed.

In contrast, in 2004, S. Boutboul et al. [8] described a family case with 24 years of follow-up suggesting a possible evolution of the plaque lesions to pseudotumor lesions.

We suggest that every patient with articular chondrocalcinosis should have an ophthalmic fundus examination to look for sclerochoroid calcification. These lesions may progress over time with possible macular involvement, thus affecting the visual prognosis. Choroidal neovascularisation is also a vision-threatening complication of sclerochoroidal calcifications that should be investigated by requesting an angiography [9].

## 4 Conclusion

Sclerochoroidal calcifications usually manifests as multiple discrete yellow placoid lesions in the midperipheral fundus of asymptomatic elderly individuals. Although they do not require treatment apart from monitoring, they should not be confused with choroidal metastasis or achromic melanoma which require more extensive treatment. Visual prognosis for sclerochoroidal classification is good, as the lesions are typically located away from the macula and foveal encroachment is rare.

## Ethical approval

This type of study does not require any ethical approval by our institution.

## Sources of funding

This study did not receive any sources of funding.

## Author contribution

O.Nabih: drafting the article, study concept, writing the article. H.Hamdani: acquisition of data. L.El maaloum: study design. B.Allali: revising the article. A. El kettani: final approval.

## Consent

Patient provided written, retrospective consent for publication following detailed explanation of the purpose of manuscript and understanding that no identifiable information was going to be released.

## Registration of research studies

Name of the registry:

Unique identifying number or registration ID:

Hyperlink to your specific registration (must be publicly accessible and will be checked):

## Guarantor

O.Nabih.

## Provenance and peer review

Not commissioned, externally peer-reviewed.

## Declaration of competing interest

The authors declare no conflict of interest.
